# The Potential Diagnostic Role of the Number of Ultrasonographic Characteristics for Patients with Thyroid Nodules Evaluated as Bethesda I–V

**DOI:** 10.3389/fonc.2014.00261

**Published:** 2014-09-23

**Authors:** Tomohiro Sakashita, Akihiro Homma, Hiromitsu Hatakeyama, Takatsugu Mizumachi, Satoshi Kano, Jun Furusawa, Satoshi Iizuka, Kimiko Hoshino, Kanako C. Hatanaka, Koji Oba, Satoshi Fukuda

**Affiliations:** ^1^Otolaryngology – Head and Neck Surgery, Hokkaido University Graduate School of Medicine, Sapporo, Japan; ^2^Surgical Pathology, Hokkaido University Hospital, Sapporo, Japan; ^3^Translational Research and Clinical Trial Center, Hokkaido University Hospital, Sapporo, Japan

**Keywords:** thyroid cancer, thyroidectomy, fine-needle aspiration cytology, ultrasound image, ROC curve

## Abstract

**Objective:** Fine-needle aspiration cytology (FNAC) is considered to be the most reliable method of examination for thyroid nodules. However, when thyroid nodules are evaluated as Bethesda I–V, the role of ultrasonography is considered to be enhanced. We investigated the association between a number of ultrasonographic (US) characteristics and the risk of thyroid malignancy, and assessed the optimal compromise on the number of US characteristics for predicting thyroid malignancy.

**Methods:** Seventy-three patients, whose thyroid nodules were evaluated as Bethesda I–V by FNAC prior to surgery, were treated surgically. A number of US characteristics, such as microcalcification, irregular margins, hypoechogenicity, a taller-than-wide shape, and the absence of halo sign, were assessed before surgery. The optimal compromise on the number of US characteristics was analyzed using a receiver operating characteristics (ROC) curve. The area under the ROC curve (AUC) represents the overall discriminatory ability of a test.

**Results:** The risk of malignancy was 11.8% in patients without any US characteristics, 44.4% in those with one characteristic, 61.5% in those with two characteristics, 75% in those with three characteristics, 90% in those with four characteristics, and 100% in those with five characteristics. The AUC was favorable (0.81599). At least two US characteristics were revealed to be the optimal compromise on the number of US characteristics based on the ROC curve.

**Conclusion:** We proved the role of the number of US characteristics in predicting thyroid malignancy. It was thought that a surgical approach should be considered for patients with at least two US characteristics.

## Introduction

Fine-needle aspiration cytology (FNAC) is considered as the most reliable method of examination by which to make decisions regarding surgical treatment for patients with thyroid nodules (1, 2). To standardize FNAC reports, the National Cancer Institute (NCI) hosted an “NCI thyroid fine-needle aspiration state of the science conference,” which led to the formation of “The Bethesda system for reporting thyroid cytopathology” (3–5). On the other hand, ultrasonography has been reported to be effective in predicting thyroid malignancies (6, 7). We believe that the role of ultrasonography is enhanced for patients with nodules that were not diagnosed definitively as malignant by FNAC. We assessed the accuracy of each ultrasonographic (US) characteristic for patients with thyroid nodules classified as Bethesda I–V. We also focused on the number of US characteristics, and determined the optimal compromise on the number of US characteristics for predicting thyroid malignancies.

## Materials and Methods

### Patients

A total of 185 patients with thyroid nodule were surgically treated between July 2010 and December 2013 in the Department of Otolaryngology-Head and Neck Surgery, Hokkaido University, Sapporo, Japan. Of these, 73 patients, whose thyroid nodules were evaluated as Bethesda I–V by FNAC before surgery, were eligible for this study. These patients consisted of 52 women and 21 men, with a median age of 58 years old (range, 24–77 years). Approval for this study was obtained from the institutional review board of Hokkaido University.

### Preoperative evaluation and ultrasonographic diagnostic criteria

Certified head and neck surgeons performed ultrasonography for patients with thyroid nodules before surgery using a HI VISION Ascendus system (HITACHI ALOKA Medical, Tokyo, Japan). Thyroid nodules were evaluated by B mode, and nodule size was recorded. US characteristics, such as microcalcification, irregular margins, hypoechogenicity, taller-than-wide shape, and the absence of halo sign, were assessed according to previous reports (6, 7). The US diagnostic criteria defined nodules as positive if any one of these five characteristics were observed (Table 1). FNAC was performed before surgery for all patients with a 21-gage needle attached to a 50 cc disposable syringe using US guidance. Two alcohol-fixed smears were prepared for Papanicolaou staining. On-site evaluation was not performed routinely. FNAC was evaluated by pathologists in accordance with the Bethesda system (3–5).

**Table 1:** **The modified ultrasonographic diagnostic criteria**.

Microcalcification	Nodules defined as positive if any one of these characteristics were observed
Irregular margins	
Hypoechogenicity	
Taller-than-wide shape	
Absence of halo sign	

### Surgical treatment and postoperative evaluation

Lobectomy or total thyroidectomy was performed based on the result of FNAC, the extent of nodules, the nodal status, or the patient’s wishes. Paratracheal nodal dissection or lateral neck dissection was added according to the nodal status. The removed thyroid nodules were assessed pathologically.

### Statistical analysis

We calculated sensitivity, specificity, positive predictive value (PPV), and negative predictive value (NPV) for each US characteristic. The risk of malignancy was also calculated according to the number of US characteristics. The optimal compromise on the number of US characteristics was analyzed using a receiver operating characteristics (ROC) curve. The ROC curve plots sensitivity against (one-specificity) for all possible thresholds in a binary classification task. The area under the ROC curve (AUC) represents the overall discriminatory ability of a test, where a value of 1.0 denotes perfect ability and a value of 0.5 denotes no ability.

## Results

### The Bethesda classification

Thyroid nodules were evaluated as non-diagnostic or unsatisfactory (Bethesda I) in 10 patients, benign (Bethesda II) in 22 patients, atypia of undetermined significance or follicular lesion of undetermined significance (Bethesda III) in 12 patients, follicular neoplasm, or suspicious for a follicular neoplasm (Bethesda IV) in 9 patients, and suspicious for malignancy (Bethesda V) in 20 patients by FNAC.

### Surgery and final pathological results

The reason for surgery was suspicious for malignancy by FNAC in 20 patients, the presence of US abnormalities in 35 patients, large size (>3 cm) in 12 patients, and the patient’s wish for a diagnostic lobectomy instead of repeat FNAC in 6 patients.

Based on final pathological results, benign lesions were observed in 34 patients, consisting of 24 cases of adenomatous goiter, 9 of follicular adenoma, and 1 of benign cyst. Malignant tumors were observed in 39 patients, consisting of one case of anaplastic carcinoma, one of poorly differentiated carcinoma, 34 of differentiated papillary carcinoma, and 3 of minimally invasive follicular carcinoma. Table 2 shows the correlation between Bethesda classification and final pathological diagnosis.

**Table 2:** **The risk of malignancy according to Bethesda classification**.

Bethesda classification	No. of patients	Final pathology		The risk of malignancy (%)
		Malignant	Benign	
I	10	6	4	60.0
II	22	8	14	36.4
III	12	5	7	41.7
IV	9	2	7	22.2
V	20	18	2	90.0

### US characteristics and diagnostic value

The median maximum nodule diameter was 22 mm (range, 5–70 mm). Microcalcification was observed in 30 patients, irregular margins in 23 patients, hypoechogenicity in 24 patients, taller-than-wide shape in 14 patients, and an absence of halo sign in 44 patients.

With the use of the modified US diagnostic criteria, 56 patients were classified as positive. In 37 of these 56 patients, malignant thyroid diseases were observed. When using diagnostic criteria, the sensitivity, specificity, PPV, and NPV were calculated as 94.9, 44.1, 66.1, and 88.2%, respectively (Table 3).

**Table 3:** **The correlation between final pathology and evaluation of the ultrasonographic (US) criteria (sensitivity 94.9%, specificity 44.1%, positive predictive value 66.1%, negative predictive value 88.2%)**.

	Final pathology		Total
	Malignant	Benign	
US Criteria
Positive	37	19	56
Negative	2	15	17
Total	39	34	73

Table 4 shows the sensitivity, specificity, PPV, and NPV for each US characteristic and tumor size.

**Table 4:** **Diagnostic value of each ultrasonographic characteristic**.

Characteristics	Sensitivity (%)	Specificity (%)	PPV (%)	NPV (%)
Tumor size >3 cm	23.1	61.8	40.9	41.2
Microcalcification	59.0	79.4	76.7	62.8
Irregular margins	51.3	91.2	87.0	62.0
Hypoechogenicity	46.2	82.4	75.0	57.1
Taller-than-wide shape	30.8	94.1	85.7	54.2
Absence of halo sign	74.4	55.9	65.9	65.5

*PPV, positive predictive value, NPV, negative predictive value*.

### The number of US characteristics and ROC curve analysis

**Figure 1:**
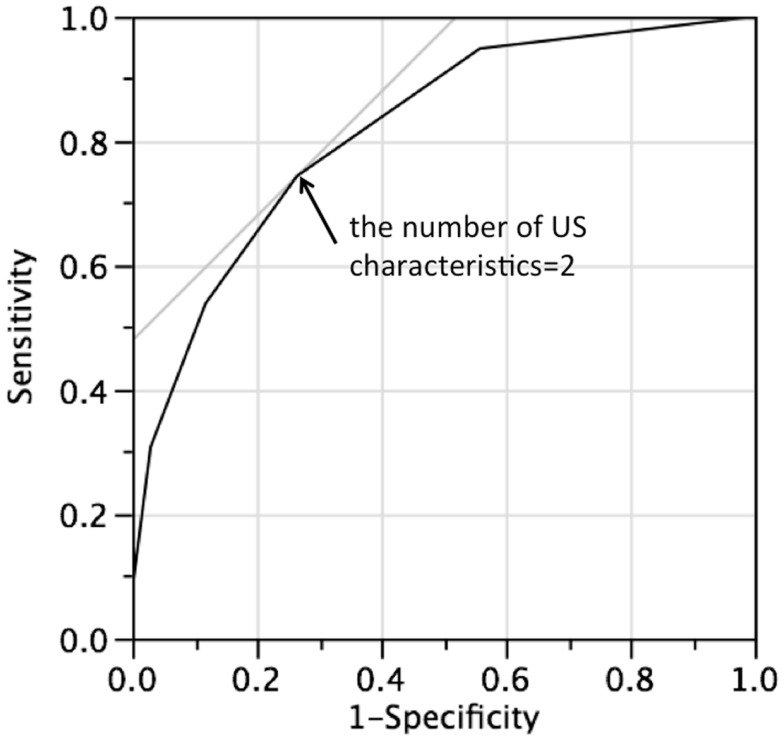
**The receiver operating characteristics (ROC) curve showing the predictive value for thyroid malignancy based on the number of ultrasonographic (US) characteristics**. The area under the ROC curve (AUC) was 0.81599.

Table 5 shows the risk of malignancy according to the number of US characteristics. Sensitivity and specificity were indicated for each number of US characteristics in Table 6. The analysis of the association between the risk of malignancy and the number of US characteristics is shown in Figure 1 with an ROC curve showing the plots of all thresholds. The value of AUC was 0.81599. At least two US characteristics was revealed to be the optimal compromise on the number of US characteristics based on the ROC curve.

**Table 5:** **The risk of malignancy according to the number of ultrasonographic (US) characteristics**.

The number of US characteristics	No. of patients	Final pathology		The risk of malignancy (%)
		Malignant	Benign	
5	3	3	0	100.0
4	10	9	1	90.0
3	12	9	3	75.0
2	13	8	5	61.5
1	18	8	10	44.4
0	17	2	15	11.8

**Table 6:** **The sensitivity and specificity of each number of ultrasonographic (US) characteristics for predicting thyroid malignancies**.

The number of US characteristics	Sensitivity (%)	Specificity (%)
5	7.7	100.0
4	30.8	97.1
3	53.9	88.2
2	74.4	73.5
1	94.9	44.1
0	100.0	0.0

### Analysis of 34 cases diagnosed with differentiated papillary carcinoma

Of 34 cases diagnosed with differentiated papillary carcinomas, five cases were evaluated as Bethesda I, seven cases were evaluated as Bethesda II, three cases were evaluated as Bethesda III, two cases were evaluated as Bethesda IV, and 17 cases were evaluated as Bethesda V. Of these 34, 3 patients had 5 US characteristics, 7 patients had 4 US characteristics, 9 patients had 3 US characteristics, 8 patients had 2 US characteristics, 6 patients had 1 US characteristic, and 1 patient had no US characteristics.

## Discussion

Both accuracy and value of FNAC for the preoperative evaluation of thyroid nodules have been established. When the cytological diagnosis is malignant, the PPV is >99%, and when it is benign, the false negative is typically <5% (4, 8). On the other hand, many authors have also found ultrasonography to be effective in predicting thyroid malignancies. Kim et al. reported the effectiveness of the US diagnostic criteria, which was defined as positive if any one of the US characteristics, such as microcalcification, irregular margins, hypoechogenicity, or taller-than-wide shape, was observed (6). They applied these criteria to patients with thyroid nodules and found that the sensitivity, specificity, PPV, and NPV were 94, 66, 56, and 96%, respectively. Rago et al. also reported that of the absence of halo sign was useful in predicting thyroid malignancies (7). In current study, we added this feature to the US criteria reported by Kim et al., and evaluated these modified US diagnostic criteria on patients with Bethesda I–V. We found that these criteria had almost the same level of accuracy as that reported by Kim et al. Although we did not find any evidence that a specific feature was particularly effective in predicting thyroid malignancies, both irregular margins and taller-than-wide shape had high PPVs (87 and 85.7%, respectively), and might be the most predictive characteristics. In addition, we were unable to prove the efficacy of nodule size in predicting thyroid malignancies. From the results of our study, we believe that nodule size should not be included in the US diagnostic criteria. The NPV of our criteria (88.2%) was satisfactory. Therefore, it might be acceptable to recommend repeat FNAC for patients without any US characteristics.

Smith-Bindman et al. suggested classifying patients with thyroid nodules according to the number of US characteristics, such as nodule size (>2 cm), microcalcification, or solid nodules (9). It was suggested that patients with <2 US characteristics have a risk of malignancy of 5 per 1000 patients, so that it was considered acceptable for biopsy or diagnostic lobectomy to be deferred in such patients. From the current study, our ROC curve analysis indicated that at least two US characteristics were optimal compromise, and we proved that the number of US characteristics was correlated significantly with the risk of malignancy, as the AUC of the ROC curve showed a favorable value (0.81599). This indicated that patients with at least two US characteristics had a risk of malignancy of 76%. Although repeat FNAC is convenient and minimally invasive, we believe that a surgical approach should be considered for patients with at least two US characteristics.

Marchevsky et al. reviewed the risk of malignancies predicted by FNAC. The rates of malignancies detected on thyroidectomy were 75% in patients with Bethesda I, 32.2% in those with Bethesda II, 37.9% in those with Bethesda III, 27.3% in those with Bethesda IV, and 100% in those with Bethesda V (10). Although our results were comparable to previous reports, it is regarded inevitable that the risk of malignancy by FNAC classification should vary quite markedly among institutions.

Ohori et al. reviewed the risk of malignancy in patients with Bethesda III, and found that the risk ratio ranged from 6 to 48% (11). Gweon et al. also reported the risk of malignancy based on thyroidectomy and/or FNAC was 70% in patients with Bethesda III. They found that the adoption of US evaluation elevated the accuracy of the diagnosis of malignancies to 85–100% (12). From these reports, it appears to be acceptable to make the decision to perform surgery on the basis of US findings for patients with thyroid nodules categorized as Bethesda III.

The limitations of this study include its retrospective nature, and the limited study population. Our study was designed to determine how to reduce unnecessary and excessive thyroid surveillance and lobectomy. Therefore, we limited inclusion eligibility to those patients undergoing surgery and with Bethesda I–V nodules. Our data might not, therefore, be applicable to every case with Bethesda I–V nodules. In addition, the accuracy of the FNAC was very low in our study. In previous reports, patients with thyroid nodules evaluated as Bethesda I–III were recommended for repeat FNAC or clinical follow-up (4, 5). However, 44 patients with nodules evaluated as Bethesda I–III underwent thyroidectomy in our study. If we undertook repeat FNAC for these 44 patients, the accuracy of the FNAC would be improved. However, we would like to recommend that the decision to undertake thyroidectomy be based on the number of US characteristics instead of repeat FNAC for patients with thyroid nodules evaluated as Bethesda I–III.

We focused on 34 cases pathologically diagnosed with differentiated papillary carcinoma. Of these 34, 12 were false negative based on FNAC findings, including 7 with Bethesda II-benign, 3 with Bethesda III-atypia of undetermined significance or follicular lesion of undetermined significance, and 2 with Bethesda IV-follicular neoplasm. After excluding five cases with Bethesda I-unsatisfactory, the false negative rate for FNAC was calculated as 41.4% (12/29). Using the criteria of at least two US characteristics, false negative findings based on the number of US characteristics were observed in seven patients (20.6%, 7/34). This focus on differentiated papillary carcinomas reconfirmed the beneficial role of the number of US characteristics due to the acceptable false negative rate.

In conclusion, we applied a modified set of US criteria, and proved the efficacy of the number of US characteristics in predicting thyroid malignancies. We believe that a surgical approach should be considered for patients with at least two US characteristics.

## Conflict of Interest Statement

The authors declare that the research was conducted in the absence of any commercial or financial relationships that could be construed as a potential conflict of interest.
